# The Value of Sequential Dermoscopy in Avoiding Unnecessary Re‐Excisions of Recurrent Nevi

**DOI:** 10.1111/ddg.70054

**Published:** 2026-01-30

**Authors:** Ina Sotiri, Federico Venturi, Elisabetta Magnaterra, Leonardo Veneziano, Aurora Alessandrini, Monika Fida, Emi Dika

**Affiliations:** ^1^ Department of Dermatology and Venereology University Hospital Center “Mother Theresa” Tirana Albania; ^2^ Department of Medical and Surgical Sciences (DIMEC) University of Bologna Bologna Italy; ^3^ Oncologic Dermatology Unit IRCCS Azienda Ospedaliero‐Universitaria of Bologna Bologna Italy

**Keywords:** digital monitoring, recurrent nevi, sequential dermoscopy

Dear Editors,

Recurrent nevi are repigmentations that develop after incomplete removal of a melanocytic nevus. Clinically and dermoscopically, they may mimic melanoma, often prompting re‐excisions and causing patient anxiety.^1^, ^2^ While dermoscopy has transformed the evaluation of pigmented lesions, a single static image may not capture the evolving morphology of a recurrent nevus.^3^, ^4^ Although only limited reports have explored the use of sequential dermoscopy to support conservative management of recurrent nevi, the role of sequential dermoscopy alone in guiding longitudinal decision‐making remains underrepresented in the literature.^5^ We present two illustrative cases where serial imaging provided reassurance and prevented unnecessary surgical intervention. In both cases, patients were referred from external dermatology clinics. Therefore, no original dermoscopic images, clinical photographs, or histologic slides were available for review. Our assessment relied on the histopathological referral reports, which confirmed the diagnosis of dermal nevus in each case.

A 46‐year‐old woman was referred for re‐excision of a recurrent pigmented macule on the lumbar region, appearing eight months after removal of a dermal nevus. Clinically, it presented as a 15 mm disarranged macule over the scar. Baseline dermoscopy revealed irregular blue‐gray pigmentation with peripheral streaks, globules, and prominent vessels (Figure 1a). Follow‐up dermoscopy at three and six months demonstrated stabilization of pigmentation, fewer globules, and no size change (Figure 1b). The overall impression, based on this sequential monitoring, was suggestive of a recurrent nevus and continued monitoring was advised.

**FIGURE 1:**
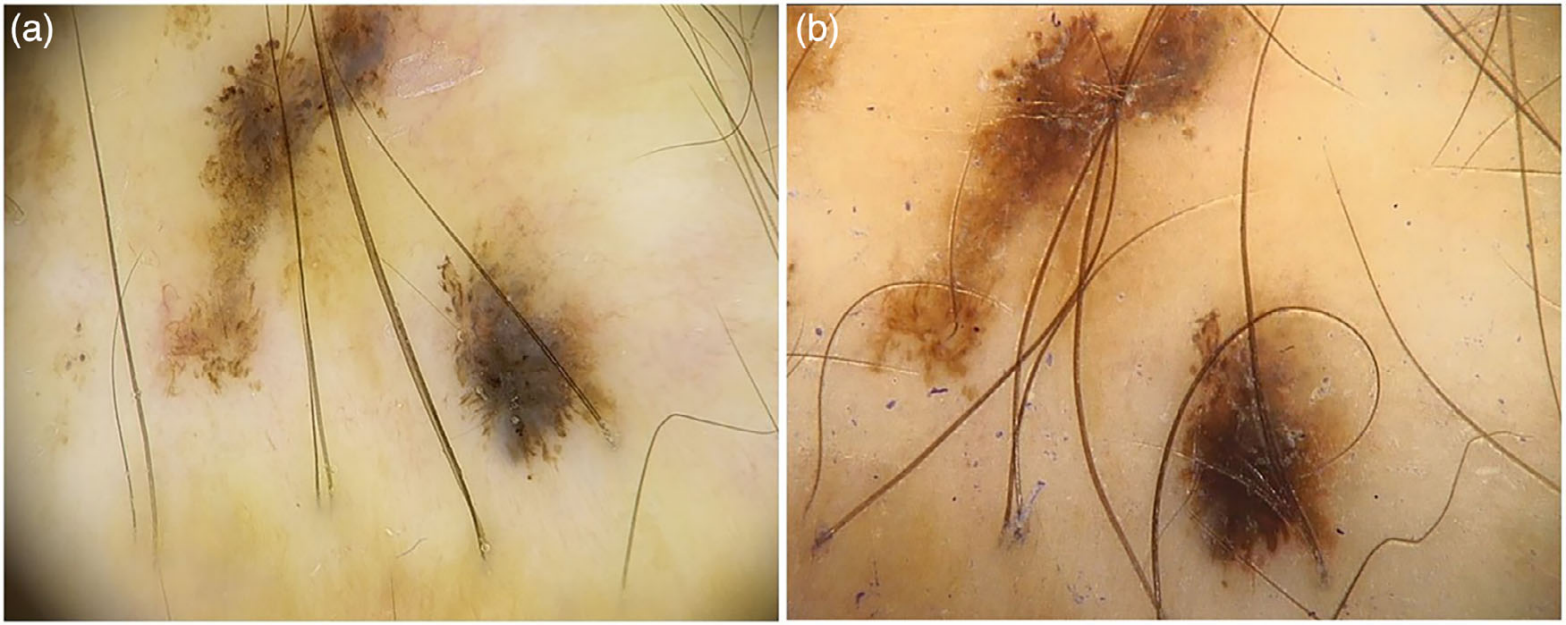
Sequential dermoscopic images of the recurrent nevus from a 46‐year‐old woman with a recurrent pigmented lesion on the lumbar region, following excision of a dermal nevus. (a) Baseline dermoscopy showing irregular blue‐gray pigmentation with peripheral streaks and globules. (b) Follow‐up at 3 months demonstrating stabilization of pigmentation, reduction of peripheral globules, and unchanged lesion size, consistent with a benign recurrent nevus.

A 48‐year‐old woman presented with two repigmented lesions on the right shoulder that developed six months after the histologically confirmed removal of dermal nevi by shave biopsy. The first lesion, a 5 mm brown macule, displayed a brown pigment network with peripheral dots and globules (Figure 2a). At three months, the lesion was unchanged, and by six months it had become smaller and more homogeneous with less prominent pigment network (Figure 2b,c). The second lesion, a 3.6 mm papule, exhibited a brown network with centrifugal growth and radial streaks confined to the scar (Figure 2d). This appearance was also concerning at first glance. By three months, pigmentation was less prominent, and by six months, the lesion had decreased in size with disappearance of streaks (Figure 2e,f). Sequential monitoring confirmed benign behaviour, avoiding two unnecessary re‐excisions.

**FIGURE 2:**
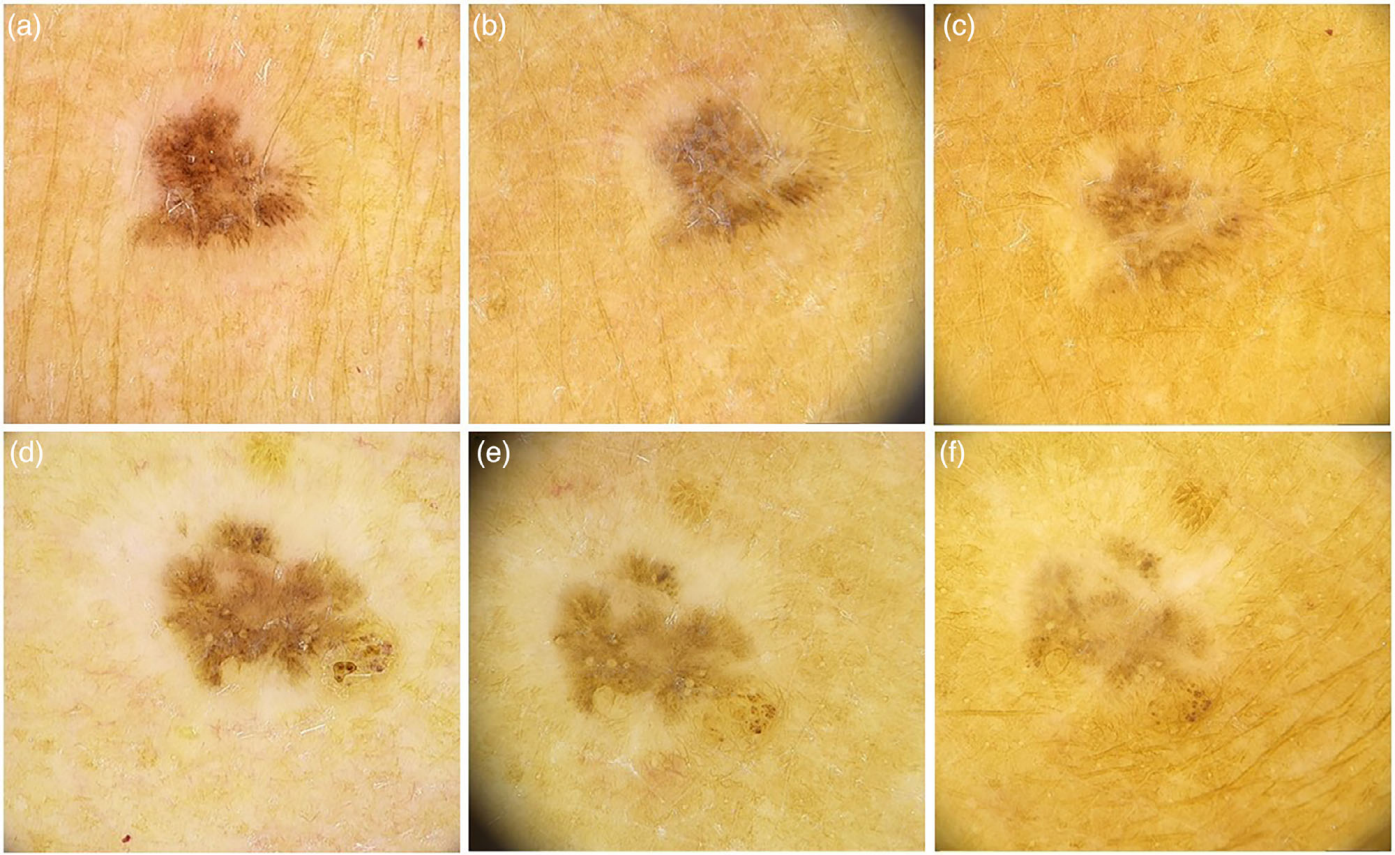
Sequential dermoscopic evolution of two recurrent nevi from a 48‐year‐old woman with two repigmented lesions on the right shoulder following excision of dermal nevi. Baseline image of a 5mm brown macule showing a brown pigment network with peripheral dots and globules (a), decreased network prominence at 3 months (b), and reduction in pigmentation with a more homogeneous pattern at 6 months (c). Baseline image of a 3.6 mm papule with a brown network and radial streaks confined within the scar (d), partial fading of pigmentation at 3 months (e), and regression with disappearance of streaks and homogeneous scar pigmentation at 6 months (f).

After six months, all lesions demonstrated stable or regressive behavior, and the patients were returned to routine annual follow‐up.

The presented cases emphasize the diagnostic challenges of recurrent nevi and underscore the clinical utility of sequential dermoscopy in distinguishing benign repigmentation from malignant recurrence. At a single time point, both lesions exhibited features commonly associated with melanoma, such as asymmetry, irregular pigmentation, and streaks.^2^ However, the temporal analysis of these patterns revealed stability or resolution of the aypical features, confirming the benign behaviour of recurrent nevi. Our findings align with prior observations suggesting that many atypical dermoscopic features in recurrent nevi result from inflammatory or reparative changes within scar tissue rather than malignant transformation.^6^, ^7^, ^8^ Most previous literature has focused on histopathology and static dermoscopy in recurrent nevi, helpful in differentiating reactive hyperpigmentation from recurrent melanocytic neoplasms, but limited in the guidance for longitudinal evaluation.^2^, ^7^ Sequential dermoscopy introduces a dynamic perspective, offering clinicians a temporal dimension to evaluate lesion behavior. This approach facilitates differentiation between active regrowth and scar‐related remodeling, helping to reduce overtreatment, surgical morbidity, and patient anxiety.^9^, ^10^, ^11^ To conclude, recurrent nevi can present with dermoscopic features that mimic melanoma, often influenced by underlying inflammatory and wound‐healing processes. Sequential dermoscopy provides a low‐cost, non‐invasive tool to monitor lesion evolution, enabling confident conservative management. In a broader clinical context, streamlined nevus assessment strategies such as estimating total body naevus count by counting naevi on the right arm can similarly aid risk stratification and decision‐making in primary care.^12^ Incorporating time‐based imaging into clinical practice may reduce unnecessary excisions and improve both diagnostic accuracy and patient outcomes.

## CONFLICT OF INTEREST STATEMENT

None.
